# Research on modification and noise reduction optimization of Electric Multiple Units traction gear under multiple working conditions

**DOI:** 10.1371/journal.pone.0298785

**Published:** 2024-02-14

**Authors:** Zhaoping Tang, Menghui Lu, Manyu Wang, Jianping Sun

**Affiliations:** 1 School of Information Engineering, East China Jiaotong University, Nanchang, China; 2 School of Transportation Engineering, East China Jiaotong University, Nanchang, China; Beijing Institute of Technology, CHINA

## Abstract

The vibration and radiation noise characteristics of the gear transmission system are different under different traction conditions, and the gear modification optimization scheme based on a single working condition is not suitable for the operating environment under all working conditions. To modify the traction gear of a high-speed EMU, an optimized design scheme for noise reduction under multiple working conditions is proposed. A modification plan of the tooth direction in conjunction with the tooth shape was devised using a parametric model of the EMU’s traction gear transmission system. The radiation noise of the gear transmission system after modification was solved using the acoustic boundary element method under different working conditions. A gear noise prediction model based on the random forest was proposed, and a gear modification parameter combination was constructed to minimize radiation noise. Then, the optimal design scheme of multi-condition modification combination parameters is obtained with the weight of the running time and acoustic contribution under different working conditions. The grey correlation degree evaluation model is established to verify that the multi-condition modification optimization design method can make the traction gear of EMU obtain satisfactory transmission performance and noise reduction effect under different working conditions.

## Introduction

Traction gear transmission systems are important components of high-speed EMUs which are Electric Multiple Units that use electricity to drive traction motors. The impact force generated by the meshing of the gears is transmitted to the casing through the shaft and bearing, forcing the casing to vibrate and resulting in the generation of radiated noise, it also causes serious damage to gears and bearings [1]. Studies have shown that radiated noise significantly affects the performance and service life of gearboxes. Therefore, it is of great significance to further explore the stability, vibration reduction, and noise reduction technology of traction gear transmission systems under multiple working conditions.

By constructing a parametric gear model, a reasonable modification design of the gear is carried out, and combining the intelligent algorithm to optimize the modification parameters is an effective way to realize vibration reduction and noise reduction of the gearbox. Yongping et al. [2] modified the gear of the planetary wheel of a planetary reducer, aiming at reducing the transmission error of gear pair meshing, improving tooth surface load distribution and contact spots, and achieving vibration reduction and noise reduction. Wang et al. [3] created a helical gear reducer simulation model using Romax, and multi-objective modification optimization was carried out by taking the gear transmission error, and peak load, and considering the tooth surface’s load distribution as the goals, realizing the noise reduction of the reducer. Wang et al. [4] modified gear modification on the high gear of the reducer of an offshore drilling platform. The results show that a reasonable modification design can effectively improve the meshing quality and reduce gear transmission error and noise. Wang et al. [5] used the orthogonal optimization method to modify the transmission for two-speed automatic transmission of pure electric vehicles and determined a reasonable modification plan. The simulation experiments demonstrated that the optimized transmission vibration and noise had a good improvement effect. TANG et al. [6] used an improved BP (Back Propagation) neural network to create a multi-stage gear transmission system noise and vibration prediction model for new-energy vehicles. The prediction model was optimized using a genetic algorithm, with minimum vibration acceleration as the optimization objective. TANG et al. [7] constructed a parameter model of the traction gear transmission system of EMU based on the analysis of the meshing characteristics of the helical gear, proposed a reasonable modification plan, and then combined it with the genetic algorithm to determine the optimal modification parameter combination. Sun et al. [8] established a planetary gear meshing stiffness-solving model based on the slice method. The effects of different modification methods and parameters on the planetary gears’ time-varying meshing stiffness were analyzed, and the optimal modification parameters of the planetary gear were obtained using a genetic algorithm. Zhou et al. [9] optimized the tooth surface of a double-stage bridge equipped with eight gears, which significantly reduced the meshing noise. Liu et al. [10] constructed a transmission simulation model using Romax by analyzing the transmission error and stiffness of the gear mesh, carrying out a comprehensive three-dimensional modification of the gear, and effectively controlling the transmission noise. Zhang et al. [11] selected a suitable plan to alter and improve the little sun wheel’s design in an electric car gearbox. The findings demonstrate that modest modifications can significantly enhance the gear meshing effect and reduce vibration and noise in the gear transmission process.

Few of the above studies directly considered the optimal noise as the optimization goal, ignoring the acoustic characteristics and noise radiation laws of the gearbox under the action of acoustic vibration coupling, resulting in a certain error between the results and the actual situation. At present, some scholars have combined the finite element and boundary element methods to analyze gearbox noise, which provides a certain basis for gearbox noise reduction. Chen et al. [12] analyzed the noise of meshing gears using finite element and boundary element methods and obtained the distribution and variation of the gear meshing noise. The simulation results were verified using sound power to determine the main excitation. Qin et al. [13] used the three-dimensional finite element nonlinear method to solve the meshing excitation of the gear, obtained the gear meshing force, and then applied the boundary element approach to account for the gear mesh radiation noise. This offers the necessary theoretical groundwork for studying gear meshing noise. Tang et al. [14] analyzed the radiated noise of the transmission system of gears using the acoustic boundary element method, built a GRNN-based radiated noise prediction model, and solved an optimization model to minimize radiation noise using the PSO algorithm. Chen et al. [15, 16] proposed an improved VICAR model and a physics-based LSTM hyperparameter selection strategy to analyze gearbox vibration signals. Wang et al. [17] established a coupled dynamic model of a secondary spur gear-shaft-bearing system under the action of multi-source time-varying excitation using the finite element method and analyzed the dynamic characteristics of the system, combined with the FEM-BEM method, and calculated and analyzed the box radiated noise. Sun et al. [18] used finite element software, multi-body dynamics analysis software, and acoustic simulation calculation software to analyze the vibration and noise problem of a gearbox and obtained an optimization scheme for the gearbox, which reduced the vibration and noise of the gearbox. Liu et al. [19] used finite and boundary elements to analyze the noise of the gearbox, solved the noise generated by the gearbox and predicted the noise radiation and noise sources of the gearbox.

Most of the existing research is based on the modification and optimization of gears under a single working condition, and based on this, the noise reduction of the gearbox is realized, ignoring the complex and changeable working conditions of the EMU. The main reason is that the vibration and radiated noise characteristics of the gear transmission system under different working conditions are different, and the simulation analysis based on a single working condition is not suitable for multiple working conditions. As a result, the method for modifying and reducing noise in traction gear systems under various working conditions proposed in this research is based on neural networks, creating a three-dimensional parametric model of the EMU traction gear transmission system, and then choosing a rational modification method. The associated vibration response characteristics of the gear transmission system were identified by a dynamic simulation analysis. The radiated noise problem was resolved using the acoustic boundary element technique, and a prediction model of the modification parameters, radiated noise based on a random forest, was constructed. The sparrow search algorithm was applied to solve the optimization model to minimize radiation noise. Based on the experimental analysis results for each working condition, the running time and acoustic contribution were used as weights to synthesize each working condition, and multi-condition modification parameters were obtained. Romax was used for unit load analysis and Vibration acceleration analysis in the NVH (Noise, Vibration, and Harshness) module in multiple working conditions, to obtain the characteristics of each node of the box under the response frequency curve and as an acoustic boundary element condition, to solve the radiated noise of the box. The necessity of the proposed method is verified by grey correlation.

## Traction gear transmission system parametric design

### Parametric modeling and load spectrum of the gear transmission system

**Fig 1:**
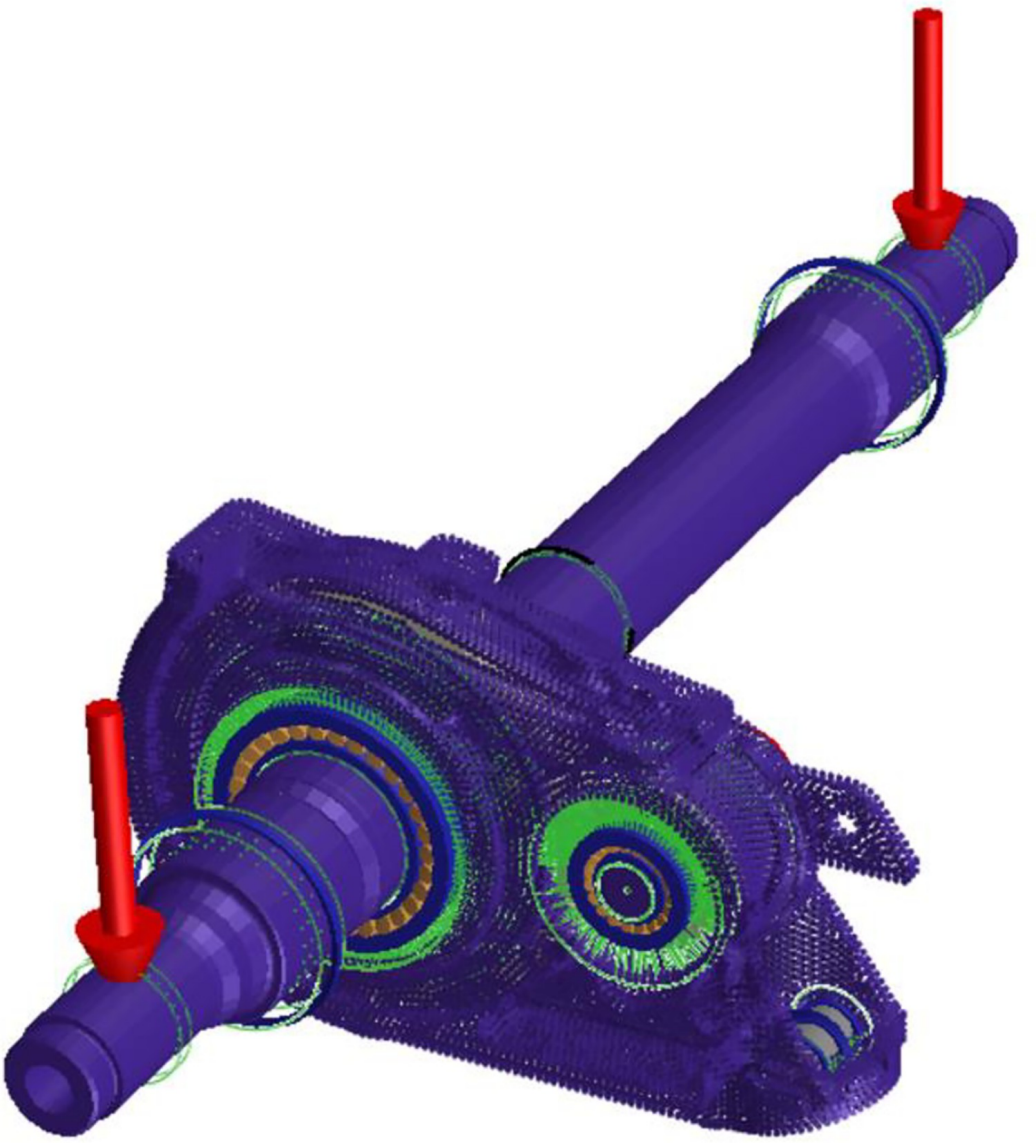
Traction gear transmission system 3D model diagram.

Taking the gear transmission system of a certain EMU as the research object, based on the model data provided by the cooperative unit, Romax software was used to establish a parametric model of the EMU traction gear transmission system, as shown in Fig 1. The specific parameters involved in the gear pair of the gear transmission system are the gear modulus 6 mm, tooth width 70 mm, pressure angle 26°, helical angle 20°, pinion tooth number 28, and large gear tooth number 85.

There are four typical working conditions in the running process EMU: starting, accelerating, continuous, and high-speed. The load spectrum parameters for each working condition during the actual operation of an interval are listed in Table 1.

**Table 1:** Load spectrum.

Working conditions	Startup condition	Acceleration condition	continuous working conditions	High speed conditions
rated power (KW)	100.5	335	300	400
Rated torque (N·M)	1200	2879	559.5	624
Rated speed (rpm)	800	3640	5120	6120
Operating time (%)	1%	5%	68%	26%

Considering space limitations, this study only selected the continuous and high-speed operating conditions of the EMU with a long-running time for evaluation, comparison, and simulation analysis. Taking pinion as an example, static analysis, contact analysis, unit load analysis, and vibration acceleration analysis of the target drive system are performed in the Romax software.

### Design of modification scheme of gear transmission system

To a certain extent, gear modification can compensate for manufacturing flaws, installation mistakes, and elastic deformation. It can also increase transmission operation stability, lower vibration, and quieter operation. To reduce gearbox vibration and noise, a reasonable gear shape modification scheme is a good option. Most of the existing modification schemes aim to improve the load distribution in the tooth direction and ignore the meshing impact caused by the tooth profile during the meshing process. Therefore, this study adopts the approach of tooth direction in conjunction with tooth profile modification to modify the driving gear, namely the pinion gear.

#### Modifying parameters for tooth direction

The tooth direction modification mainly includes drum shape modification, spiral Angle modification, and tooth end thinning. When the gear is in a bad tooth direction meshing state, a series of adverse effects such as uneven distribution of tooth direction load and serious load bias will be generated. Through drum modification, the load can be evenly distributed in the middle of the tooth surface to reduce impact and noise [20]. Drum shape modification is easy to process, the design is simple, and the quality of the modification is easy to control. Although the calculation of the modification amount of tooth end thinning is not complicated, the application range of tooth end modification is small and the modification effect is not obvious. The effect of spiral Angle modification is more obvious, but the amount of Angle adjustment is difficult to determine and the processing cost is high. When the machining Angle is too small, it is very difficult to modify the shape and the effect is no advantage compared with other methods, and the spiral Angle modification is not suitable for mass production operations. Therefore, to reduce the processing cost and improve efficiency, the practicability and cost-benefit of the design are weighed, this paper mainly focuses on drum shape modification.

The most crucial factor in drum shape trimming is the amount of the shape, and it has a direct bearing on the effect of trimming as well as the bearing capacity of the shape after trimming. If the drum volume is too large, the tooth surface will produce a high contact stress, resulting in more accelerated gear wear and failure to repair the effect of the shape. If the drum volume is too small and cannot play a role in compensating for tooth meshing errors, the same will cause load concentration. Choosing an appropriate drum size is crucial for reducing the gearbox noise. Based on the ISO 6336–1 criterion for calculating the amount of drum modification, the empirical reference value is combined with the calculation formula for the modification of the drum shape in Eqs (1) and (2):

$$C_{a}=0.25B\times 10^{-3}+0.5f_{g}$$ (1)

$$f_{g}=A(0.1B+10)$$ (2)

where *C*_*a*_ represents the volume of drum shape (μm); *B* represents tooth width (mm); *f*_*g*_ indicates the accuracy determined tooth error(μm); *A* stands for the coefficient for which the accuracy class is determined. Gear accuracy is mainly the accuracy that controls the transmission between gears when the gear is running. Considering the overall consideration, the gear accuracy level is set to 6 when the model is constructed in this paper. The relationship between *A* and accuracy level is shown in the following Table 2, the value of is taken as 2.0 at the level 6 accuracy.

**Table 2:** The relation table between gear grade accuracy coefficient and A.

accuracy	0	1	2	3	4	5	6	7	8
A	0.63	0.71	0.8	1	1.25	1.6	2.0	2.5	3.15

#### Parameters for modifying the tooth profile

Top trimming and root trimming are commonly used techniques for modifying gear tooth profiles. The main idea is to use appropriate methods to modify the gear teeth. surface material in the direction of the tooth profile to reduce the impact on meshing that results from the collision between the tooth top and tooth root when the gear meshes. Subsequently, the load distribution on the tooth surface should be optimized to lower the likelihood of vibration.

In addition to being influenced by the elastic deformation of the gear teeth under load, the base pitch deviation during production also affects the amount of tooth profile alteration that occurs. This formula is expressed as follows:

$$\Delta =\delta \pm \Delta f_{b}$$ (3)

where Δ indicates the degree of modification of the tooth profile (μm), *δ* indicates the elastic deformation of the gear under a load (μm), and Δ*f*_*b*_ indicates the base pitch inaccuracy during machining (μm).

The gear’s elastic deformation as measured using GB/Z6413.1–2003 is:

$$\delta =\frac{K_{A}K_{\mathrm{mp}}F_{t}}{B\cos\alpha_{t}c_{r}}$$ (4)

where *K*_*A*_ represents the gear’s working condition’s coefficient of usage, *K*_*mp*_ stands for branching factor; *B* means tooth width (mm), *α*_t_ is the terminal face’s pressure angle, *F*_*t*_ indicates the tangential force (N), and *c*_*γ*_ stands for engagement rigidity (N/(mm·μm)). Here *K*_*A*_ and *K*_*mp*_ were taken as 1.

The following formula can be used to express the nominal tangential force of the gear transmission:

$$F_{t}=\frac{2000T}{d}$$ (5)

where *F*_*t*_ stands for the tangential force (N); *T* stands for nominal torque (N·m); *d* indicates the gear indexing circle’s diameter (mm).

According to the parameters of the traction gear transmission system, it can be known torque *T* = 559.5 (N·m) under continuous working conditions, torque *T* = 624 (N·m) under high-speed working conditions; Index circle diameter *d* = 178.78 (mm); End pressure Angle *α*_t_ = 27.43°; The meshing stiffness *c*_*γ*_ = 19.54 (N/(mm·μm)) and the machining pitch error Δ*f*_*b*_ = 7 (μm) were calculated by inserting parameters, and the maximum profile modification of the pinion under continuous and high-speed working conditions was 12.16μm and 12.75μm.

#### Determination of the scope of modification

Based on the small number of teeth, small size, large deformation, and other factors of the small and medium gear and the selection of gear modification mode, the pinion tooth direction modification volume, tooth inclination and tooth profile modification amount are selected as the modification parameters. In this paper, the tooth direction and tooth profile modification parameters of EMU traction gear are calculated by Eqs (1–5). However, the maximum modification amount obtained by the theoretical calculation formula cannot be accurately determined as the optimal modification amount. To seek the optimal modification amount below, the sampling range of modification is set, As shown in Table 3:

**Table 3:** The parameter’s value range for the shape alteration.

	Continuous working condition	high speed condition
Modification parameters name	Ranges (μm)	Ranges (μm)
Amount of tooth repair	0–17	0–17
Tooth inclination	-17–17	-17–17
Amount of tooth profile modification	0–12.160	0–12.75

## Simulation analysis of traction gear drive system

Before making modifications, dynamically simulate the gear transmission system model while taking unit load analysis, transmission error analysis, and acceleration analysis for box vibration into consideration. An acoustic numerical simulation of the gear transmission system was performed using the acoustic boundary element method, and the magnitude of the radiated noise was solved. Prepare for the next modification and offer a foundation for effect comparison for modification optimization.

### Unit load analysis

An essential indicator for assessing the impact of a noise reduction model is gear unit load analysis, which directly reflects the contact state between gears. The gear transmission system is more stable when the load is uniformly distributed. The gear unit load is along the tooth contact lines per unit length of the average load and is expressed as:

$$O=\frac{F_{n}}{L}$$ (6)

$$F_{n}=\frac{F_{t}}{\cos\varpi }$$ (7)

where *F*_n_ refers to the average force applied to the tooth surface’s contact line (N), and *L* is along the tooth surface contact line length (mm), *F*_*t*_ is obtained from Formula (5), and *ϖ* is the helix angle.

**Fig 2:**
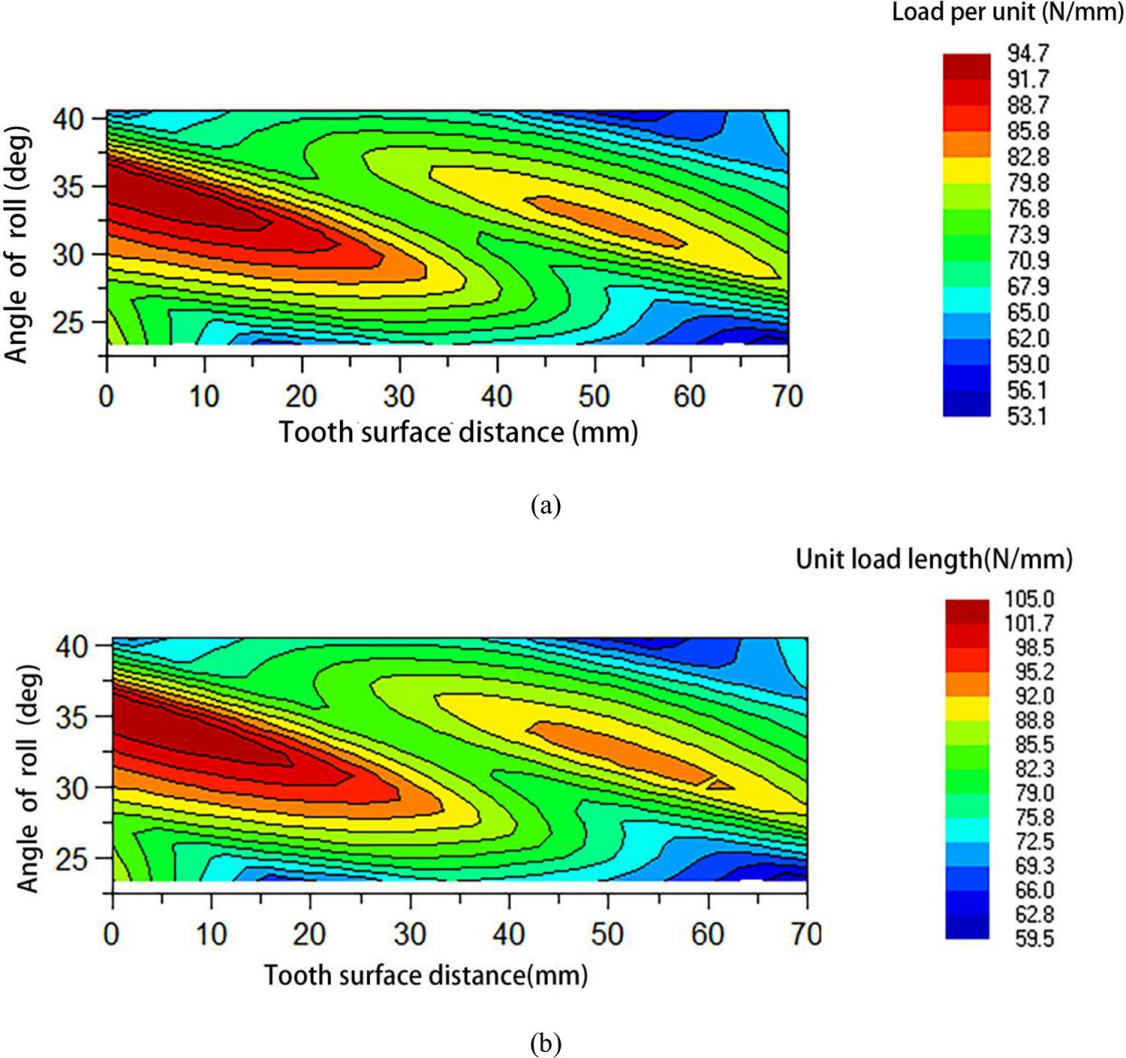
(a) Analysis of cloud diagram of pinion unit length load under continuous working condition; (b) Analysis of cloud diagram of pinion unit length load under high-speed11 condition.

Based on the unit load theory of the above gears, this paper applies corresponding loads to the gear transmission system through the gear tooth contact analysis module in the Romax software under continuous and high-speed working conditions, and obtains the unit load through numerical calculation, which is represented by Fig 2.

Analyzing Fig 2, under continuous and high-speed conditions, the contact locations of the gear sets reveal that for both large and small gears, the left side receives more stress load than the right side. The results demonstrate that an offset load develops during the meshing of the gear pair and that this offset load readily reduces the stability of the gear transmission and raises vibration noise. The subsequent modifications should achieve a uniform load.

### Transmission error analysis

Gear transmission error refers to the difference between the peak values of the transmission error curve, and it has a considerable impact on the gear transmission system’s vibration and noise. Following is the theoretical formulation:

$$TE=\varepsilon \gamma_{b2}‐\theta_{1}\gamma_{b1}$$ (8)

where *TE* stands for transmission error, *γ*_b1_ indicates the driving wheel circle radius for pitch, *γ*_b2_ represents the driven wheel pitch circle radius, *θ*_1_ is the driving wheel angle of theoretical transmission, *ε* and is the driving wheel true transmission angle.

Owing to the actual situation, there are installation errors, and the influence of the meshing impact, the true drive angle of the driving wheel is:

$$\varepsilon =\theta_{2}+\Delta \theta_{2}$$ (9)

where *θ*_2_ represents the theoretical drive angle of the driven wheel, and Δ*θ*_2_ represents the angular departure value due to transmission error.

**Fig 3:**
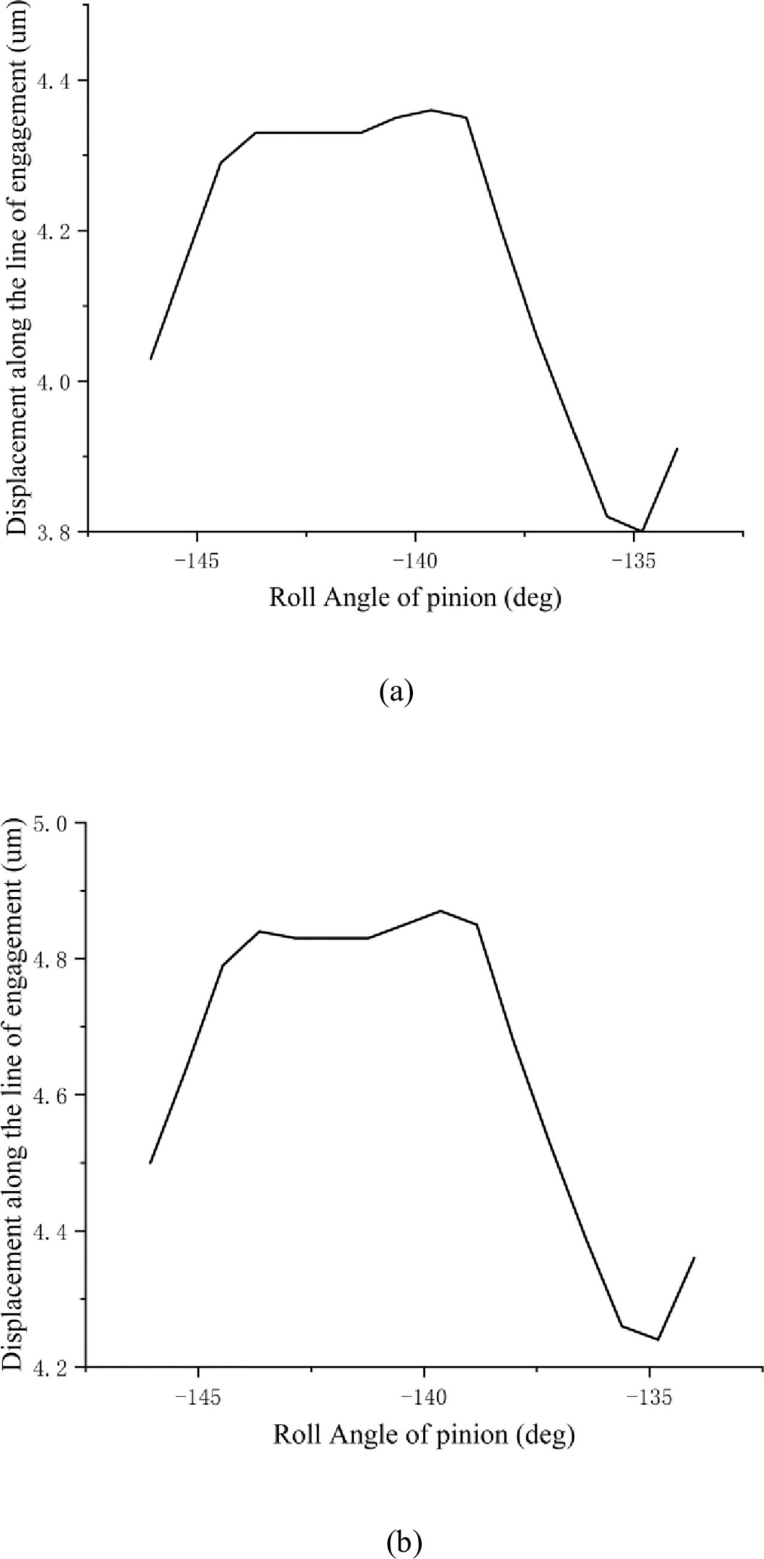
(a) Transmission error under continuous working conditions; (b) Transmission error under high-speed conditions.

Under sustained and high-speed conditions, using Romax software, the examination of the EMU transmission system’s transmission error simulation was performed, and a 2D drawing report of the gear set transmission linearity error results is obtained, as shown in Fig 3, Table 4 shows the data table for transmission linearity error:

**Table 4:** Gear set transmission linearity error data.

	Continuous working condition		high speed conditions	
	Roll Angle of pinion 1 (deg)	Displacement along meshing line (μm)	Roll Angle of pinion 1 (deg)	Displacement along meshing line (μm)
maximum value	-134.010	4.36	-134.010	4.87
Minimum value	-146.063	3.80	-146.063	4.24
scope	12.054	0.5628	12.054	0.6262

By analyzing the above charts, it can be found that the transmission errors of the gears in the continuous working and high-speed working conditions are 0.5628 μm and 0.6262 μm, respectively. To fulfill the goal of minimizing noise, the subsequent gear adjustment should lower the transmission error.

### Vibration acceleration analysis

NVH stands for Noise, Vibration, and Harshness [21] and is used to analyze the noise and vibration characteristics of a mechanical system. In the gear transmission system, the vibration acceleration of the box is the main excitation source of radiation noise, which has a great influence on the noise of NVH performance. Calculated for each working situation is the transmission gear’s vibration response and the gear transmission system’s noise response characteristics are analyzed. The system’s equation for undamped forced vibration is:

$$\left[M]\{\ddot{u}\}+[K]\{u\}=\{F\right\}$$ (10)

where **u** indicates the displacement of a vibration; $\ddot{u}$ represents the acceleration of vibration.

The following is an expression for the harmonic response’s excitation load:

$$F_{i}=F_{i\max}\sin(\sigma t+\beta_{i})$$ (11)

where *F*_*i*max_ represents the amplitude of the load, *σ* indicates the load frequency, *β* and represents the load phase angle.

**Fig 4:**
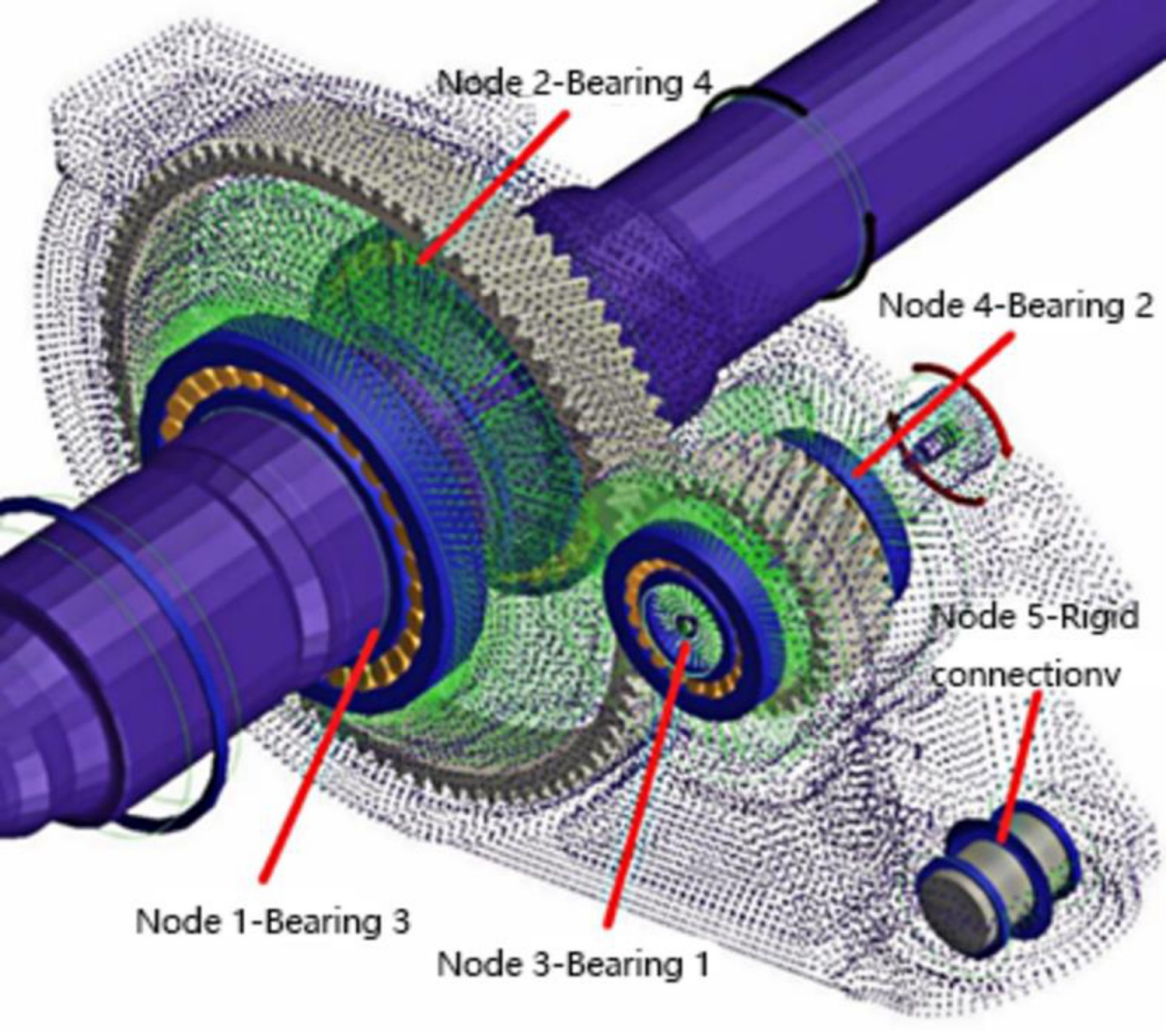
Node location diagram of gear transmission system.

**Fig 5:**
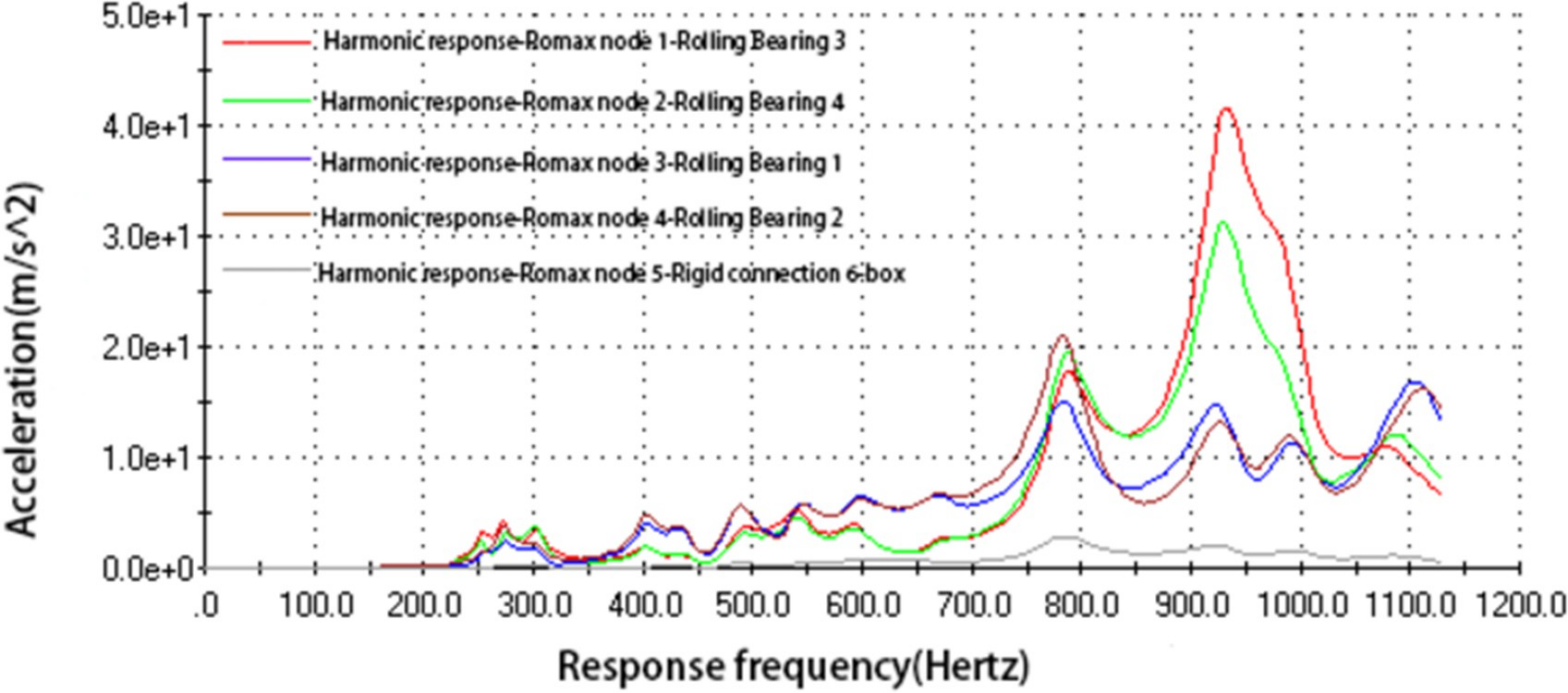
Each node box’s vibration acceleration curve.

Since the noise of the gear transmission system is studied in this paper, and the noise generated by gear meshing is mainly reflected in the box, of which the largest noise is reflected in the bearing and the rigid connection of the gearbox. In order to analyze the vibration of the entire gearbox, we select 5 nodes with the largest vibration characteristics and use them to verify the shape modification effect, namely, Node 1 (rolling bearing 3), Node 2 (rolling bearing 4), located at the output shaft; node 3 (rolling bearing 1), node 4 (rolling bearing 2), located at the input shaft; node 5 (rigid connection) as shown in Fig 4. Under the stimulation of the gear meshing transmission error, the amplitude curves corresponding to the vibration acceleration and frequency of the five nodes on the gearbox are shown in Fig 5.

Each node’s characteristics are revealed in the box beneath the response frequency curve in Fig 5. Node 1 (rolling bearing 3) and node 2 (rolling bearing 2) correspond to larger vibration acceleration at 930 Hz, indicating that the vibration noise generated by the two nodes is the most obvious. The results of this analysis can be applied as excitation conditions to obtain data on the vibration response of the gearbox.

### Noise in gear transmission system numerical analysis

By getting the structure’s surface mesh, the acoustic radiation was calculated using the boundary element approach, which is based on the Helmholtz boundary integral equation. Describes the gear transmission system’s vibration noise that reverberates through the air. The Helmholtz BEM equation for the acoustic system can be expressed as follows:

$$p={\left[A_{\nu }(w)\right]}^{T}\nu_{n}(w)$$ (12)

where *p* indicates the sound pressure, *w* denotes the associated frequency, *A*_*v*_(*w*) represents the sound delivery vector, and *v*_*n*_(*w*) indicates the structural surface element’s normal velocity. Where T stands for *A*_*v*_(*w*) transpose.

In any location in the gear transmission system’s radiated sound field that is w distance from the sound source, the sound pressure *p* and normal velocity *v*_*n*_ fulfill the following equations:

$$p(r_{a})=\sum_{i=1}^{n_{e}}N_{i}^{e}(r_{a})\cdot a_{pi},r_{a}\in \Omega_{ae}$$ (13)

$$v_{n}(r_{a})=\sum_{i=1}^{n_{e}}N_{i}^{e}(r_{a})\cdot a_{vi},r_{a}\in \Omega_{ae}$$ (14)

where *n*_*e*_ is the quantity of Ω_*ae*_ nodes of a given cell, *a*_*pi*_ is the sound pressure on a boundary element cell’s node, and *a*_*vi*_ is the normal velocity on the cell’s node. $N_{i}^{e}$ is the form function of a cell.

This paper mainly discusses the radiated noise generated by the EMU system of gear transmission and how gear modification can be used to achieve noise reduction. To assess the size of the noise, sound power was used as the acoustical quantity. For ease of comparison, the sound power level’s root mean square (RMS) values at different frequencies were used as the calculation result for the acoustic simulation.

The average sound energy per unit of time passed perpendicular to the plane is called the average sound power. This can be expressed as follows:

$$W=\xi c_{0}S$$ (15)

In the formula, *c*_0_ represents the pace at which sound travels; *S* is the region in which sound waves diffuse; *ξ* indicates the acoustic energy density, and is calculated as follows:

$$\xi =\frac{E}{V_{0}}=\frac{1}{2}\rho_{0}(v^{2}+\frac{1}{2\rho_{0}c_{0}^{2}}p^{2})$$ (16)

where *E* is the total energy of the sound wave; *v* is the vibration velocity (m/s); *V*_0_ indicates the acoustic energy unit’s initial volume, *p*_0_ indicates the initial pressure, *ρ*_0_ and indicates the density. The sound level of power can be stated as follows to allow for comparison:

$$L_{\omega }=10log\frac{W}{W_{0}}$$ (17)

In the formula: reference sound power *W*_0_ = 10^−12^*W*.

**Fig 6:**
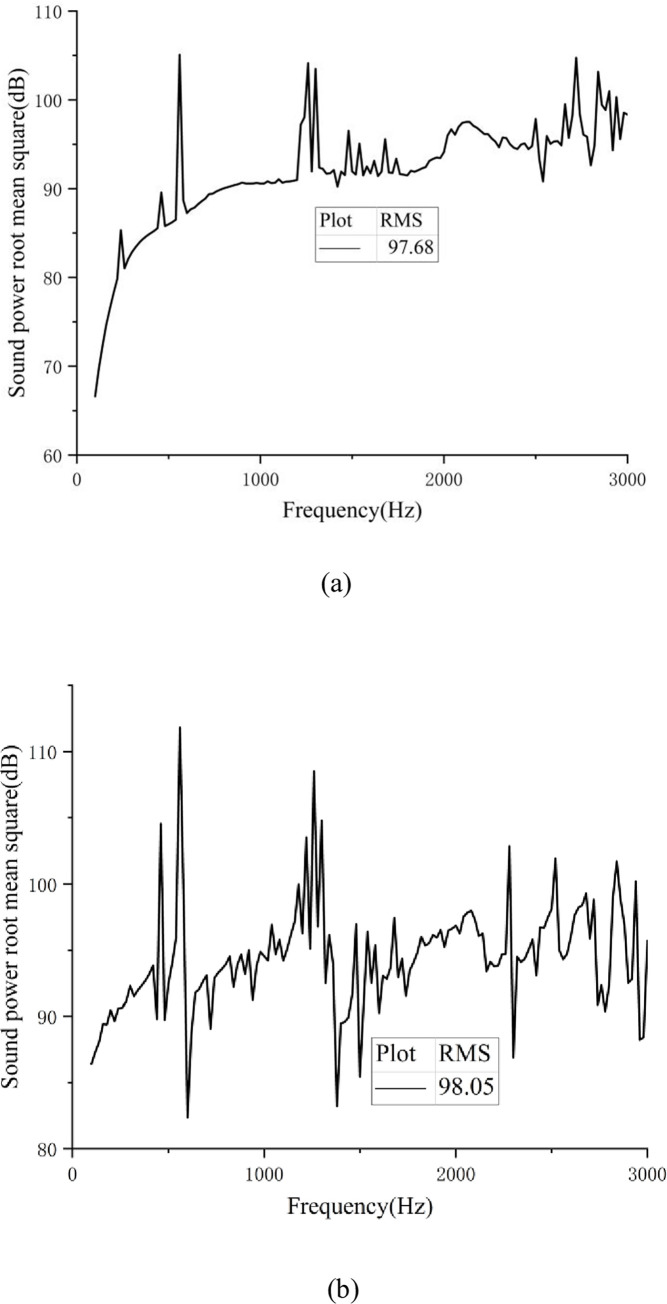
(a) Curve of sound power under continuous working condition; (b) Sound power curve under high-speed condition.

For the surface acoustic simulation study, the gearbox finite element model and vibrational-responsive data were imported into the virtual environment. Laboratory acoustic boundary-element module. Subsequently, the noise of the gearing system was calculated [22]. Combination of Eqs (15–17), the radiated noise sound power level curve, and the root mean square (RMS) of the sound power level of the gear transmission system under continuous working conditions and high-speed working conditions can be obtained, as shown in Fig 6.

According to the acoustic power curve, the root mean square (RMS) of the radiated noise of the EMU gear transmission system under continuous working conditions was 97.68 dB. The root mean square (RMS) of the radiated noise of the EMU gear transmission system at high speed was 98.05 dB. These data offer a comparable basis for subsequent modification and optimization.

## Optimization design of traction gear transmission system based on random forest

The complicated nonlinear properties of the gear transmission system make it difficult to converge to the best theoretical solution, which necessitates a significant computing workload for the optimal design of a complex nonlinear system. In contrast to the conventional linear regression method, random forest can completely reflect the benefits of data mining because it does not require making assumptions about the function form beforehand, which minimizes assumption errors. The mapping function of the gear modification parameters—radiation noise—was constructed in this study using a random forest. Then the sparrow search algorithm is utilized to solve for the ideal modification amount of the gear pair under each working state, resulting in the ideal modification parameters.

### Random forest

A random forest is a collection of categorization decision trees that have not been pruned and generated algorithmically. The forest output is determined by simple majority voting or by taking the simple average of each tree’s output. The classification model is mostly targeted by a simple majority voting approach, whereas the regression model is primarily targeted by the simple average of a single tree’s output findings [23].

The random forest regression model employs non-linear regression, assuming that an in the model comprises *M* tree $\left\{T_{1}(X),T_{2}(X),\ldots ,T_{M}(X),\right\}$. where *X* = {*x*_1_,*x*_2_,…,*x*_*P*_} is the forest’s *p*-dimensional input vector. Each tree generates a value that is predicted, ${\hat{Y}}_{1}=T_{1}(X),\ldots ,{\hat{Y}}_{M}=T_{M}(X)(M=1,\ldots ,M)$. To obtain the model’s output result, all trees’ prediction values are finally averaged. This is the specific procedure:

1. The bootstrap approach is applied to create a decision tree using a set of self-help samples that are randomly chosen, with the test set always consisting of the unsampled data.
2. During decision tree development, a feature is randomly selected from all the characteristics at each node, and a feature is then chosen from this feature for branch growth by the principle of lowest node impurity. The nodes of the decision tree continue to separate and grow entirely by the same rule, and until the branch-stop criterion is satisfied and growth stops, each node’s impurity should be kept to a minimum.
3. Predict the required data using many decision tree classifiers that have been constructed, and then calculate the average regression result using the predictions from each decision tree.

The training set for each regression tree was randomly chosen from the original dataset to construct the random forest regression model. The prediction accuracy of the model was checked using a test set created from the data that were not used for modeling. Random’s capacity for generalization is significantly enhanced by its built-in verification function.

### Extraction of modified parameter variables

The sampling range of each modification parameter obtained in Table 3 was sampled, and 200 sets of modification parameter combination data were sequentially sampled for continuous and high-speed working conditions using the Insight software’s best Latin hypercube sampling module.

**Fig 7:**
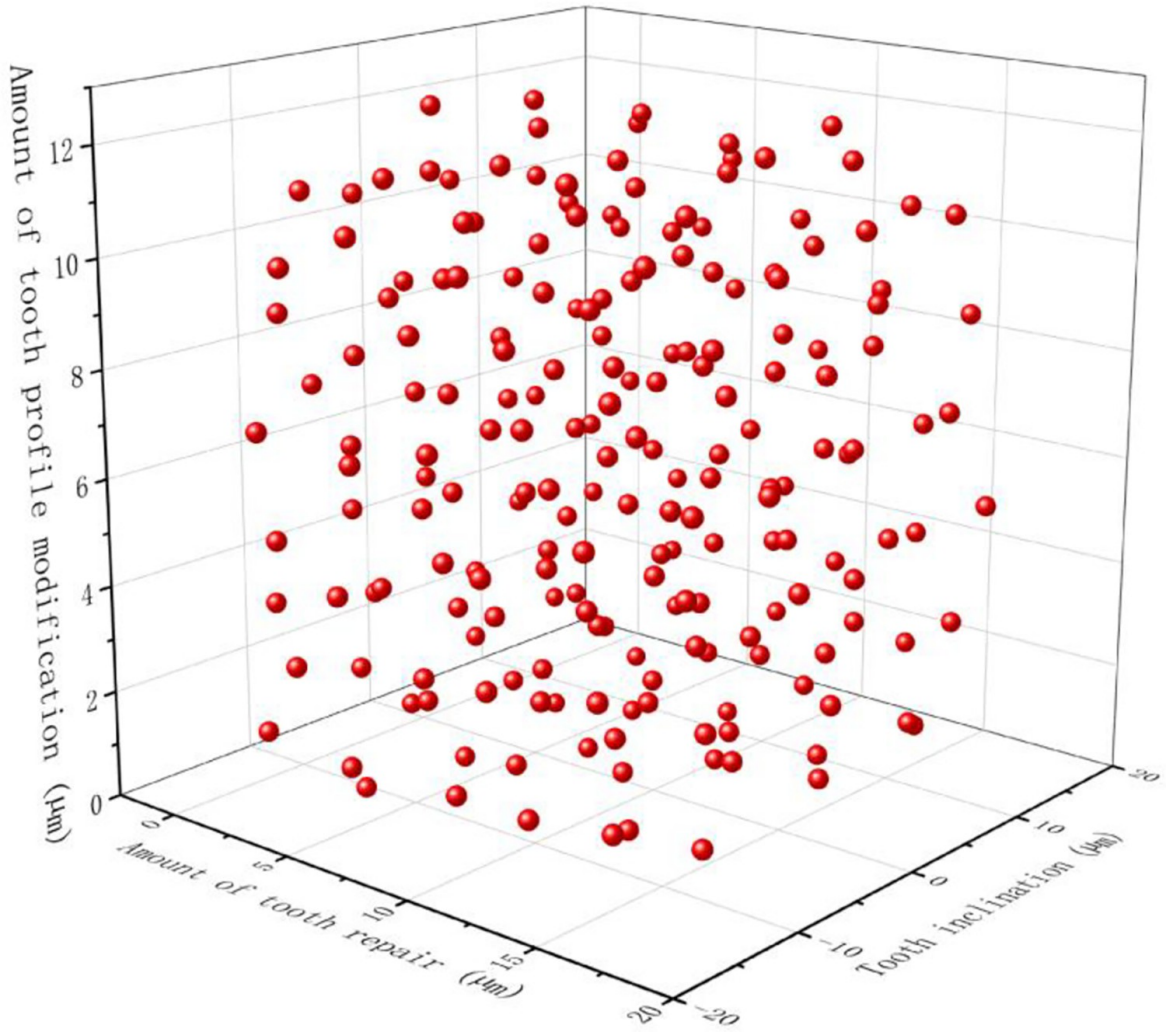
Data distribution in the OptLHD sampling scheme.

Fig 7 shows the spread of the combinations of the parameters of the modified shape in the area of the samples. As can be seen, the data selected using the optimal Latin superelevation approach can be distributed evenly throughout the sample area.

### Constructing a gear system for transmission noise prediction model based on random forests

The modification parameter sets acquired through sampling under the two working conditions are combined to build the gear modification model in Romax software in turn, and the dynamic simulation analysis and acoustic simulation analysis are carried out to obtain the noise data corresponding to each modification parameter.

The simulation-derived noise data were collected and the corresponding noise-to-modification-parameter relationship was created, as indicated in Table 5.

**Table 5:** Correlation of modification parameters with noise in each working condition(part).

	Continuous working condition				high speed working condition			
serial number	Amount of tooth repair (μm)	Tooth inclination (μm)	Amount of tooth profile modification (μm)	RMS simulation sound power level value (dB)	Amount of tooth repair (μm)	Tooth inclination (μm)	Amount of tooth profile modification (μm)	RMS simulation sound power level value (dB)
1	3.417	15.97	2.566	93.09	9.312	-9.65	7.24	97.93
2	13.412	7.26	0.489	90.89	14.352	5.89	5.51	85.41
3	13.754	0.77	11.977	89.75	2.307	-16.49	9.867	103.34
4	3.673	10.34	9.471	95.20	9.995	1.62	3.78	83.50
5	3.075	8.97	5.622	83.55	13.839	-12.9	9.803	97.03
6	2.307	-16.83	7.149	100.16	16.06	6.75	3.332	87.94
7	12.643	-7.77	0.428	97.98	7.176	-0.6	4.357	83.46
8	12.985	5.04	4.766	85.23	2.819	10.17	7.753	89.23
9	2.819	-10.68	6.538	98.04	7.347	-11.02	5.446	98.08
10	8.457	12.05	5.866	83.41	16.658	1.45	5.638	85.92
	⋮	⋮	⋮	⋮	⋮	⋮	⋮	⋮
200	6.92	-13.58	9.105	101.90	15.719	5.04	9.675	86.98

To further explore the correspondence between the EMU traction gear modification parameters and radiated noise, the input data for the prediction model consisted of 200 sets of gear modification parameters sampled under each working state. The prediction model’s output data was the radiation noise for each modification parameter combination, and an EMU’s traction gear transmission system noise prediction model was built.

In terms of the model, the output of the neural network model was chosen to be the gear transmission system’s radiated noise, and three modification parameters were chosen as inputs, building the radiated noise prediction model:

$$z=f(C_{a},l,\Delta )$$ (18)

where *z* is the root mean square of the sound power level of the associated scanning frequency range of the gear transmission system’s radiated noise (dB); *C*_*a*_ is the pinion tooth direction drum repair (μm); *l* is the slope of the pinion tooth (μm); Δ is the amount of modification to the pinion tooth profile (μm).

**Fig 8:**
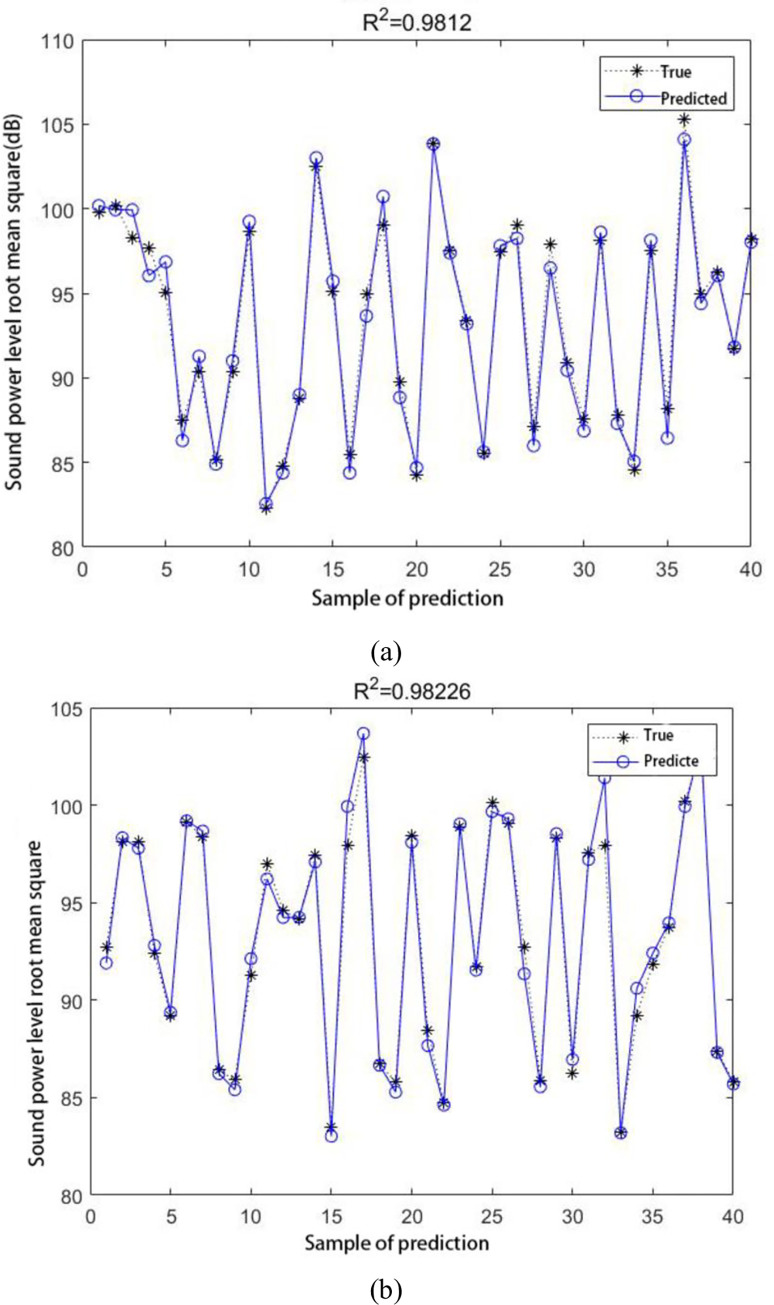
(a) Random Forest fitting effect graph under continuous working conditions;(b) Random Forest fitting effect graph under high-speed conditions.

The specific parameters of the random forest are set to bootstrap: true, max_depth: 50, max_features: auto, min_samples_leaf: 2, min_samples_split: 2, n_estimators: 500, we chose a random 80 percent of the modification parameter-noise data combination [24]. Additionally, 20 percent of the data were used to verify the model. The fitting effects of the random forest neural network test set on the data under continuous and high-speed working conditions are shown in Fig 8.

Generally, when the fitting mass R^2^ is greater than 0.9 and closer to 1, the prediction model has a higher fitting effect [25]. As shown in Fig 8, the goodness of fit of the model under continuous working conditions reached 0.9812, and the goodness of fit of the model under high-speed working conditions reached 0.98226. The gear modification parameters of the radiation noise dataset are shown to be well-fitted by the optimization model, suggesting that the prediction model can correctly anticipate the noise.

### Model optimization with the sparrow search algorithm

Comparatively speaking to other swarm intelligence optimization algorithms, the Sparrow search algorithm has fewer parameters, strong stability, fast convergence speed, high search accuracy, and strong global search ability [26].

In this paper, sparrow search algorithm is introduced to solve the optimization model of traction gear transmission system. The objective function of the optimization model is the RMS value of the minimum radiated noise sound power level, and the constraint condition is the solution space formed by the three modified parameter variables. The objective function is shown in Eq (19), and the constraint condition formed by the parameter range is shown in Eq (20):

$$\min z=f(C_{a},l,\Delta )$$ (19)

$$s.t.\{\begin{array}{l} 0\leq C_{a}\leq 17\\ ‐17\leq l\leq 17\\ 0\leq \Delta \leq 12.160 \end{array}$$ (20)

The optimization model is solved using the sparrow search algorithm, which performs the essential steps in parameter optimization as follows:

The parameters of the sparrow search algorithm were initialized [27]. Since there were three modification variables in this paper, the dimension of the problem was set to 3. To prevent overcalculation, the population size was set to 20, and the number of iterations was set to 50. Fitness function values were calculated and sorted. By dividing the population into finders and followers, the proportion of discoverers is set to 20%, the proportion of followers is set to 10%, a guard is randomly selected to continuously update the position for optimization, and the prediction results of the random forest prediction model are used as the initial value of the fitness function to search for the optimal gear modification parameter data. The traction gear modification parameters’ optimal outcome was attained, as indicated in Table 6.

**Table 6:** Optimal solution of pinion modification parameter combination under various working conditions.

working condition	Amount of tooth repair (μm)	Tooth to the slope (μm)	Amount of tooth profile modification (μm)	Sound power level prediction value (dB)
Continuous working condition	4.8	7.74	5.21	81.59
high speed conditions	6.71	14.71	5.93	81.74

## Multi-working condition modification and noise reduction design

Additional requirements for gear optimization are now included in gear optimization design. In terms of traction gear optimization design, people want the vibration and noise reduction of the gearbox can be achieved by changing the gear shape under a single working situation, and they also hope that the effect can be better under several working conditions. However, high-speed EMU operating circumstances are complicated and varied, making it challenging to build a system that considers numerous working conditions and meets user expectations based on gear optimization based on a single working state. As a result, the running time ratio of each working condition and the gearbox sound power level was added as indicators to weigh the weight ratio and determine the optimal gear modification parameters under various working conditions based on the idea of obtaining the optimal gear modification parameters under a single working condition. Grey relational degree analysis was proposed to analyze the modification scheme to confirm the accuracy of the multi-condition modification. Considering the limitation of space, this study only considered two typical operating conditions of the EMU, continuous operating conditions and high-speed operating conditions, as examples to carry out the optimization design research of multi-operating condition modification.

### Determination of multi-working modification and noise reduction scheme

In Romax software, the model for gear modification was created, and the analyses of the dynamic and acoustic simulations were carried out. Acquire noise data, build a noise prediction model solve it using the sparrow search algorithm, and obtain the multi-stage gear modification parameter combination’s best solution under continuous and high-speed working conditions. The sound contribution is obtained by integrating the sound power level of the radiated noise of the gearbox under each working condition and the corresponding timeshare, and the optimal modification parameter combination under the weighted comprehensive continuous working condition and the high-speed working condition is obtained, and the modification parameter combination of multiple working conditions is obtained, the formula is as follows:

$$S=w_{1}(t_{1}A_{1}+t_{1}A_{2})+w_{2}(v_{1}A_{1}+v_{2}A_{2})$$ (21)

Formula: *S* represents the combination vector of multi-condition modification parameters, *A*_1_ represents the combination quantity of modification parameters under continuous condition, *A*_2_ represents the combination quantity of modification parameters under high-speed condition, *t*_1_ and *t*_2_ respectively represents the proportion of running time of high-speed EMU under continuous condition and high-speed condition, *v*_1_ and *v*_2_ respectively represent the proportion of acoustic contribution under continuous and high-speed condition, *w*_1_ represents the weight value of time proportion. *w*_2_ represents the acoustic contribution weight value.

It can be seen from Table 1 that the running time of high-speed EMU under continuous and high-speed conditions accounted for 68% and 26% respectively. It can be seen from Table 5 that the modification parameters under continuous and high-speed conditions were [4.8,7.74,5.21] and [6.71,14.71,5.93], respectively. According to the noise solution process in Chapter 3, the radiated noise under various working conditions is obtained as [97.68,98.05,99.6,101.5], *v*_1_ and *v*_2_ are calculated by calculating the proportion of total radiated noise under various working conditions under continuous and high-speed working conditions. Considering that the objective of this paper is mainly for the design of vibration and noise reduction of EMU and through discussion with railway experts, the acoustic contribution is ranked first, the weight ratio of acoustic contribution is 0.6, and the weight ratio of time contribution is 0.4. The combination of multi-working condition modification parameters was obtained by substituting the numerical values into the calculation, as shown in Table 7.

**Table 7:** The optimal solution of the combination of pinion modification parameters under multi-working conditions.

Amount of tooth repair (μm)	Tooth to the slope (μm)	Amount of tooth profile modification (μm)
3.71	6.96	3.68

### Multi-condition modification noise reduction effect analysis

**Fig 9:**
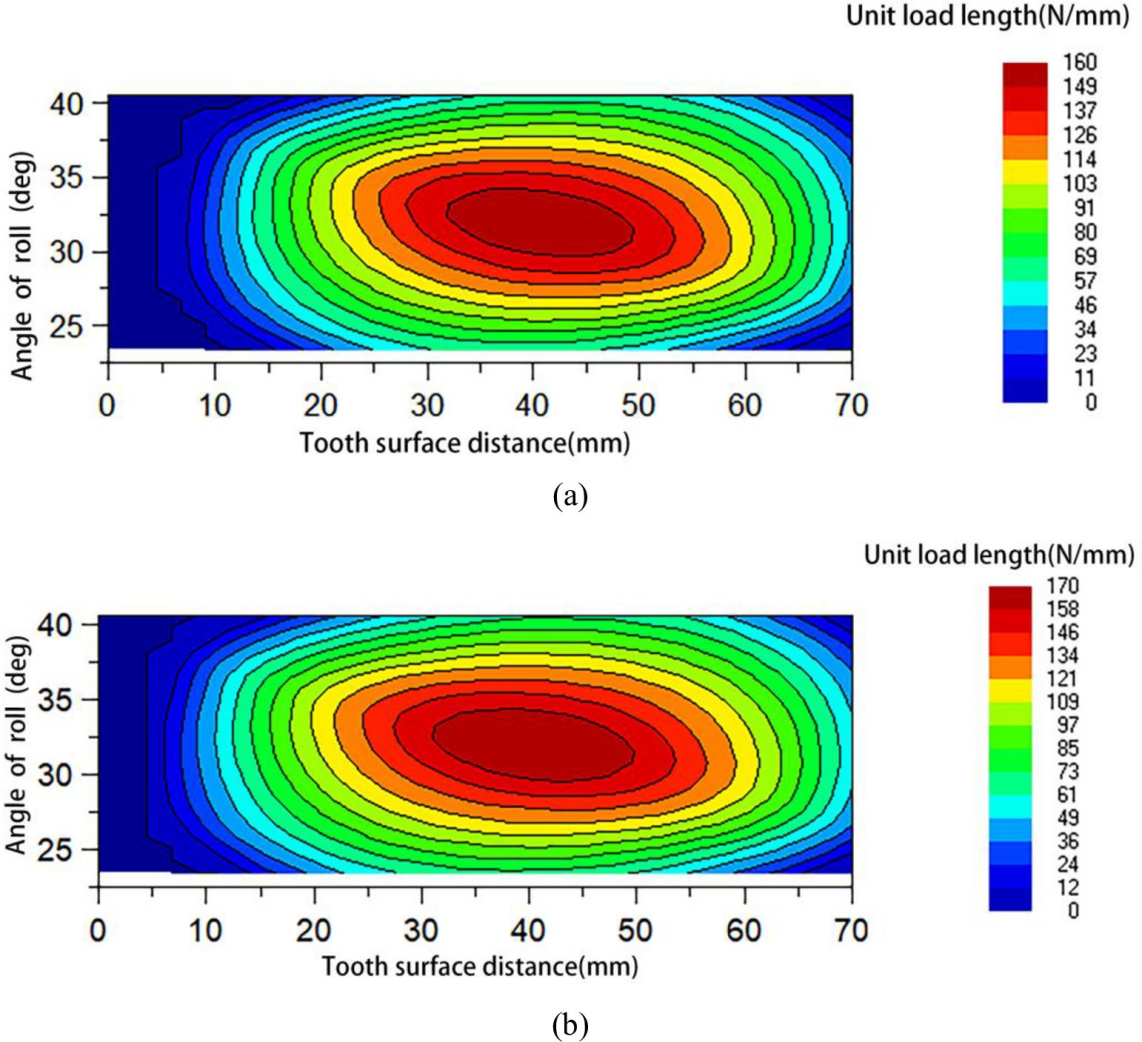
(a) Unit load distribution of multi-working modification parameter under continuous working conditions. (b) Unit load distribution of multi-working modification parameter under high-speed conditions.

**Fig 10:**
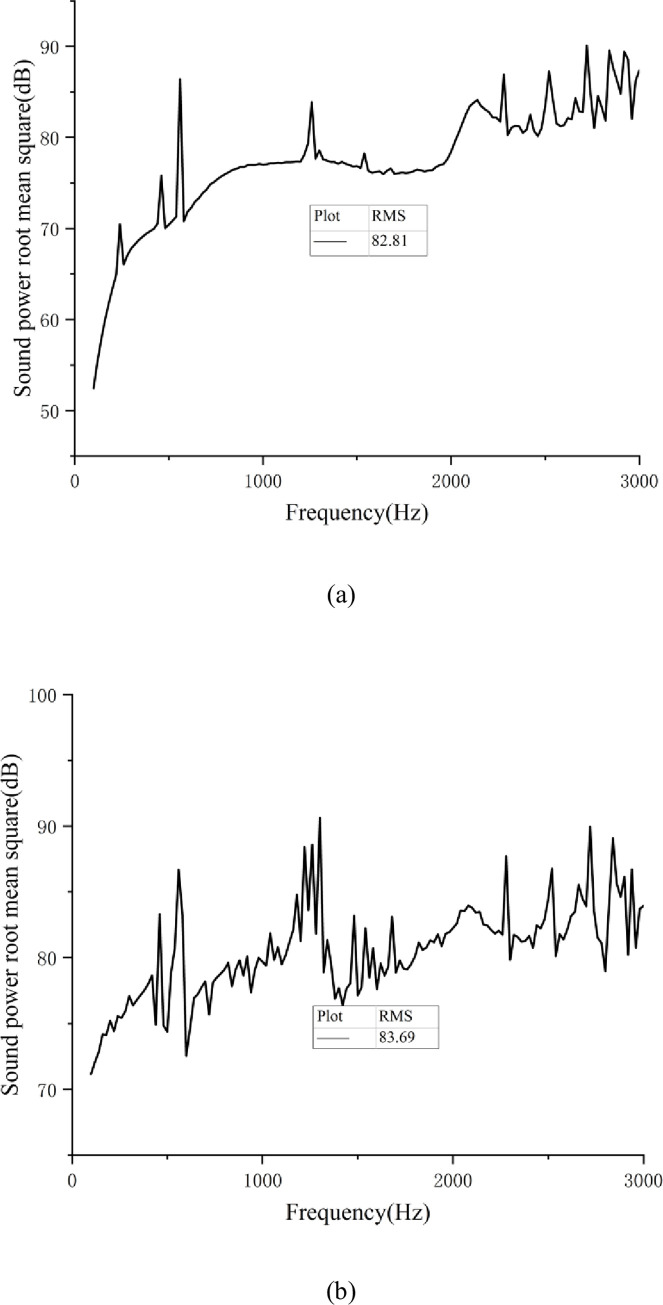
(a) The minimum sound power level of multi-working modification parameter under continuous working conditions. (b) The minimum sound power level of multi-working modification parameter under high-speed conditions.

The RMS value of the sound power level of the gearbox is an important embodiment of the noise reduction effect of the gearbox, and the gear transmission error and unit load distribution are key indicators for measuring the vibration and noise reduction effect of the gear transmission system. A series of simulation analyses were performed on the multi-condition optimal gear modification parameters under continuous and high-speed conditions. The gearbox radiation noise was solved using the acoustic boundary element method, and the distribution of the gear unit load and sound power level corresponding to the optimal gear modification parameter set under multi-working conditions were obtained, as shown in Figs 9 and 10. Table 8 summarizes the simulation results.

**Table 8:** Simulation analysis data before and after modification comparison.

	Continuous working condition		high speed conditions	
	Before the modification	After the modification	Before the modification	After the modification
Transmission Error (μm)	0.563	0.2266	0.626	0.1782
Unit load distribution	Severe eccentric load	even distribution	Severe eccentric load	even distribution
Sound power level RMS value (dB)	97.68	82.81	98.05	83.69

Summarizing the simulation analysis results and the comparison before modification, it can be seen that the modified design of multi-working conditions can achieve a uniform distribution of gear load distribution and improve the gear bearing capacity. Under continuous working conditions, the gear pair’s transmission error amplitude is decreased by 0.3364 μm, a reduction of 59.6%, and the sound power level is reduced from 97.68 dB to 82.81 dB, a drop of about 15.2%. A decrease of 0.4478 μm in the magnitude of transmission error of the gear pair under high-speed conditions, a decrease of 71.5%, and the sound power level is reduced from 98.05 dB to 83.69 dB, a reduction of about 14.6%. The findings demonstrate that the modification parameters of multiple working conditions can successfully lower the gear transmission system’s radiated noise, ameliorate the gear’s unit load distribution, and significantly reduce the transmission error of the gear pair.

### Grey correlation analysis method

To verify that the multi-condition modification has a superior modification effect compared to the single-condition modification. The grey correlation analysis approach is presented in this study to thoroughly assess the modification effect of the three modification target schemes. Grey correlation analysis uses the degree order of grey correlation to characterize the direction, magnitude, and strength of the relationships between various components. It is a technique for analyzing and determining the extent to which each system factor affects other factors or how much each factor contributes to the system’s primary behavior [28]. The following actions were taken.

1) Assuming that m targets have n solutions, the target eigenvalue matrix is expressed as:
$$\left(x_{1}^{'},\cdots ,x_{n}^{'})=(\begin{array}{cccc} x_{1}^{'}(1) & x_{2}^{'}(1) & \cdots & x_{n}^{'}(1)\\ x_{1}^{'}(2) & x_{2}^{'}(2) & \cdots & x_{n}^{'}(2)\\ \vdots & \vdots & \ddots & \vdots \\ x_{1}^{'}(m) & x_{2}^{'}(m) & \cdots & x_{n}^{'}(m) \end{array}\right)$$ (22)
  In the formula, *m* stands for the number of indicators; *n* represents the quantity of modification plans.
2) We are determining the reference data sequence. Select the appropriate reference value from the optimal value of each indicator to form a reference series. Notated as:
$$x_{0}^{'}=[x_{0}^{'}(1),\cdots ,x_{0}^{'}(m)]$$ (23)
3) Dimensionless quantization of metrics. Before grey correlation analysis, Formula (24) was used to dimensionless the index data.
$$x_{i}(j)=\frac{x_{i}^{'}(j)}{\frac{1}{m}\sum_{i=1}x_{i}^{'}(j)}$$ (24)
4) Find two levels of maximum difference and two-level minimum differences. Calculate the difference in absolute value between the data of each assessed object and the relevant reference sequence item, as in Formula (25), and to find the two-level maximum contrast and the two-level minimum difference are Eqs (26) and (27), respectively.
$$\Delta_{i}(j)=|x_{0}(j)-x_{i}(j)|$$ (25)
$$\Delta (\max)=\overset{n}{\underset{i=1}{\max}}\;\overset{m}{\underset{j=1}{\max}}\;\Delta_{i}(j)$$ (26)
$$\Delta (\min)=\overset{n}{\underset{i=1}{\min}}\;\overset{m}{\underset{j=1}{\min}}\;\Delta_{i}(j)$$ (27)
5) Calculation of correlation coefficient. Calculate the correlation coefficient corresponding to each index series and the reference series respectively, as shown below:
$$\gamma_{i}(j)=\frac{\Delta (\min)+\psi \cdot \Delta (\max)}{\Delta_{i}(j)+\psi \cdot \Delta (\max)}$$ (28)
  where *ψ* is the resolution factor, 0<*ψ*<1,the difference between the correlation coefficients is greater and the ability to discriminate is stronger if *ψ* is smaller. Generally, *ψ* takes the value of 0.5.
6) Determine the static eigenvalue of the node. The average correlation coefficient between each index sequence and the relevant components of the reference sequence was computed, and this measurement is known as the degree of association, to reflect the correlation between each assessment object and the reference sequence. It can be written as a Formula (29):
$$\gamma_{0i}=\frac{1}{m}\sum_{i=1}\gamma_{i}(j)$$ (29)

### Evaluation value solution

Based on the analysis of each index related to the gear dynamics performance, the dimensionless processing of each index is carried out. The correlation degree of the correlation coefficient corresponding to each index series is calculated. Finally, the best solution is determined according to the correlation degree.

Many factors affect the dynamic performance of the gear. To facilitate comparison, three indexes of transmission error, maximum load per unit length, and sound power level of gearbox radiated noise were selected for evaluation. Let the target set by:

$$U=\{u_{1},u_{2},u_{3},u_{4},u_{5},u_{6}\}$$

where *u*_1_ is the transmission error amplitude under continuous working condition; *u*_2_ is the maximum load per unit length under continuous working condition; *u*_3_ is the noise radiated by the gearbox under continuous working condition; *u*_4_ is the transmission error amplitude under high speed condition; *u*_5_ is the maximum unit length load under high speed condition; *u*_6_ is the noise radiated by the gearbox under high-speed condition. Let the modification scheme set by:

$$V=\{h_{1},h_{2},h_{3}\}$$

where *h*_1_ is the continuous optimal modification parameter combination; *h*_2_ is the combination of high-speed optimal modification parameters; *h*_3_ is a combination of modification parameters.

The three modification schemes were subjected to a series of simulation experiments, and the modification effect assessment data table for each combination of the modification parameters was obtained, as displayed in Table 9.

**Table 9:** Summary table of indicators for each modification parameter combination.

Program Indicators	Option 1 (continuously optimal)	Option 2 (high-speed optimal)	Option 3 (multi-condition optimal)
Transmission error under continuous working condition (um)	0.1303	1.05	0.2266
RMS value of sound power under continuous working conditions (dB)	80.549	90.07	82.81
Transmission error at high speed (um)	0.9834	0.1158	0.1782
RMS value of sound power at high speed (dB)	92.39	80.7	83.69
Unit load under continuous working Conditions	141	205	160
Unit load at high-speed working condition	194	145	170

The reference sequence is determined by the optimal value of each index:

$$x_{0}^{'}=[0.1303,80.54,0.1158,80.7,141,145]$$

Perform dimensionless processing on the indicators, find the two-level maximum difference and the two-level minimum difference, then the correlation coefficient *γ*_*i*_(*j*) is solved for each index series corresponding to the reference series by Eq (28):

$$\gamma_{i}(j)=[\begin{array}{ccc} 1 & 0.34 & 0.84\\ 1 & 0.9 & 0.97\\ 0.33 & 1 & 0.87\\ 0.87 & 1 & 0.96\\ 0.77 & 1 & 0.86\\ 0.82 & 0.83 & 0.9 \end{array}]$$

According to Formula (29), the *γ*_0*i*_ of each scheme is solved as the evaluation result of each modification parameter combination. As shown in Table 10.

**Table 10:** Summary of evaluation results of each modification parameter combination.

Program	Option 1 (Continuously optimal)	Option 2 (High-speed optimal)	Option 3 (Multi-condition optimal)
Evaluation Results	0.83	0.83	0.9

## Conclusion

This paper takes the optimization of traction gear modification and noise reduction of high-speed EMU as the research objective, fully considers the variable operating conditions of EMU and the dynamics and acoustic characteristics of the gear transmission system, and designs a multi-operating condition modification scheme of gear tooth direction combined with tooth profile direction. By using the finite element and boundary element method, a prediction model of gear transmission radiation noise based on random forest is proposed. A modified optimization model aiming at minimizing noise is constructed and solved by a sparrow search algorithm. Taking the gear shape optimization effect as an index system, a grey relational degree evaluation model was established, and a multi-condition shape optimization design scheme of EMU was proposed. The outcomes indicate that the unit load distribution of the gears after the modification is greatly improved, and the gear pair transmission error is significantly decreased under different traction conditions. Among them, the radiated noise of the gearbox under continuous working conditions is reduced by 14.87 dB, a decrease of 15.2%;14.36 dB lower gearbox radiation noise under high-speed conditions, a decrease of 14.6%. The grey correlation degree evaluation model is established to verify that the overall modification effect of the multi-condition comprehensive modification scheme has certain advantages. The main research results are as follows:

1. In this paper, a proposed multi-condition modification approach for gear tooth direction and tooth profile. The finite element and boundary element method is used to import the modified traction gear transmission system model through dynamic simulation, and the excitation force obtained is imported into LMS Virtual. Lab. The optimal Latin hypercube sampling is used to sample the gear modification parameters, and the random forest neural network is used to construct the prediction model of the modification parameter combination-radiation noise, which can more accurately and intuitively reflect the radiation noise intensity of the traction gear transmission system under different modification parameter combinations.
2. Using random forest neural network to build modification parameter-radiation noise prediction model. Solving a modified-parameter optimization model with radiation noise minimization as the goal using the sparrow search algorithm and the optimal modification parameter combination under different traction conditions is obtained.
3. An optimization design method of gear modification parameters under multi-working conditions is presented. With the weight of running time and acoustic contribution under different working conditions, the optimal design scheme of multi-working conditions modification combination parameters is obtained. With the optimization effect of gear modification as the index, the grey correlation degree evaluation model was established to verify that the multi-condition modification optimization design method can ensure that the traction gear of EMU can obtain satisfactory transmission performance and noise reduction effect under different working conditions.

Aiming at the noise reduction requirements of the traction system of high-speed EMU under multi-operating conditions, this paper puts forward the modified optimization design of traction gear and simulates its dynamic characteristics and sound radiation noise by using the boundary element method, which theoretically verifies the feasibility of the model and method. The future research direction will also focus on the real working environment of the traction gear transmission system, and continue to explore the multi-parameter combined gear modification scheme of the gear to further optimize the dynamic contact performance of the gear transmission and reduce the radiation noise of the gear transmission. In addition, referring to the previous research results of loading test ideas and methods [29] of the class group and cooperative units on the repair effect of locomotive current traction gear, the simulation analysis results of this paper are further verified by experiments.
